# Supplementary material for: Easy-to-use nomogram to predict neonatal hyperbilirubinemia
Source: PeerJ. 2025 Sep 3;13:e20017. doi: 10.7717/peerj.20017 (PMC12422276; doi:10.7717/peerj.20017)
Supplement: Supplemental Information 1 [file peerj-13-20017-s001.docx]

FIGURE 1


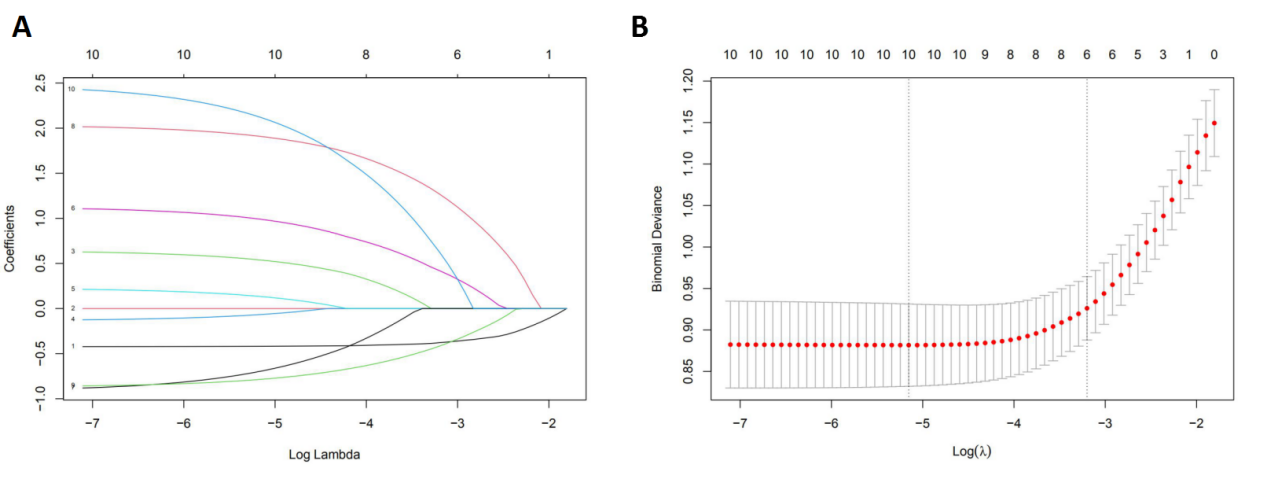


| > rm(list = ls(all = TRUE))  > ####Work Path Setting  > setwd("C:/Users/23983/Desktop/R results")  > ####Data import  > #It is recommended to use UTF-8 csv format to import data  > data <- read.csv("trainTotal data (+ disaggregated information) (10 risk factors).csv",header = TRUE)  > ##########LASSO regression screening data predictors  > library(glmnet)  > set.seed(214825)  > #Two pieces of data needed to build LASSO  > x = data.matrix(data[,c(3:12)])  > y = data.matrix(data[,c(2)])  > #Conducting LASSO  > fit = glmnet(x,y,family = "binomial",alpha=1)  > #alpha=1 lasso regression, alpha=0 ridge regression.  > fit  > plot(fit,xvar="lambda",label=TRUE)  > #Cross-validation  > cv.fit <- cv.glmnet(x, y, family="binomial",nfolds = 10)  > cv.fit  plot(cv.fit)  > abline(v=log(c(cv.fit$lambda.min,cv.fit$lambda.1se)),lty=2) |
| --- |
|  |
