# Supplementary material for: Easy-to-use nomogram to predict neonatal hyperbilirubinemia
Source: PeerJ. 2025 Sep 3;13:e20017. doi: 10.7717/peerj.20017 (PMC12422276; doi:10.7717/peerj.20017)
Supplement: Supplemental Information 2 [file peerj-13-20017-s002.docx]

| FIGURE 2  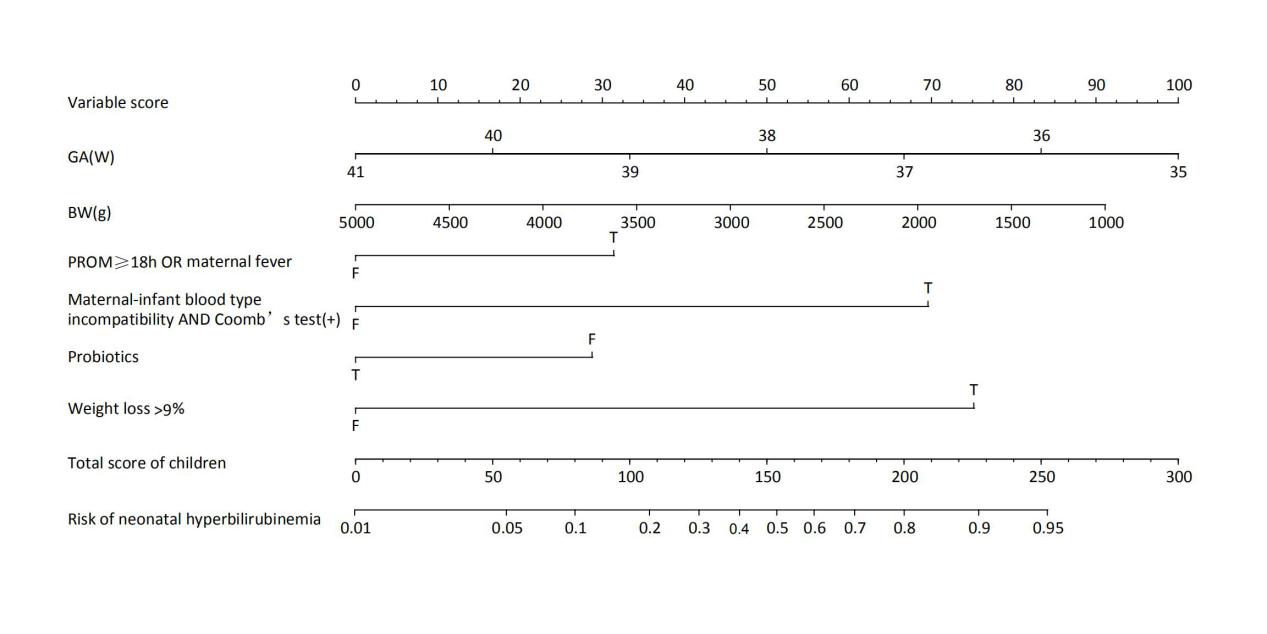  > rm(list = ls(all = TRUE))  > ####Work Path Setting  > setwd("C:/Users/23983/Desktop/R results")  > ####Data import  > #It is recommended to use UTF-8 csv format to import data  > data <- read.csv("trainTotal data (+ disaggregated information) (10 risk factors).csv",header = TRUE)  > library(rms)  > dist.train <- datadist(train)  > options(datadist="dist.train")  > fit.full <- lrm(END ~ V2_num+V3_num+V8+V17+V20+V21,  + data = train,x = T,y = T)  > #Line Chart Generation  > nom.ss <- nomogram(fit.full,fun=plogis, fun.at=c(.001,.01, .05,  + seq(.1,.9, by=.1), .95, .99, .999),lp=F,  + funlabel="Risk of neonatal hyperbilirubinaemia")  > #Line drawing  > plot(nom.ss,vnames='labels',points.label = "Variable score",total.points.label = "Total score of the children") |
| --- |
