# Supplementary material for: Easy-to-use nomogram to predict neonatal hyperbilirubinemia
Source: PeerJ. 2025 Sep 3;13:e20017. doi: 10.7717/peerj.20017 (PMC12422276; doi:10.7717/peerj.20017)
Supplement: Supplemental Information 3 [file peerj-13-20017-s003.docx]

| FIGURE 3  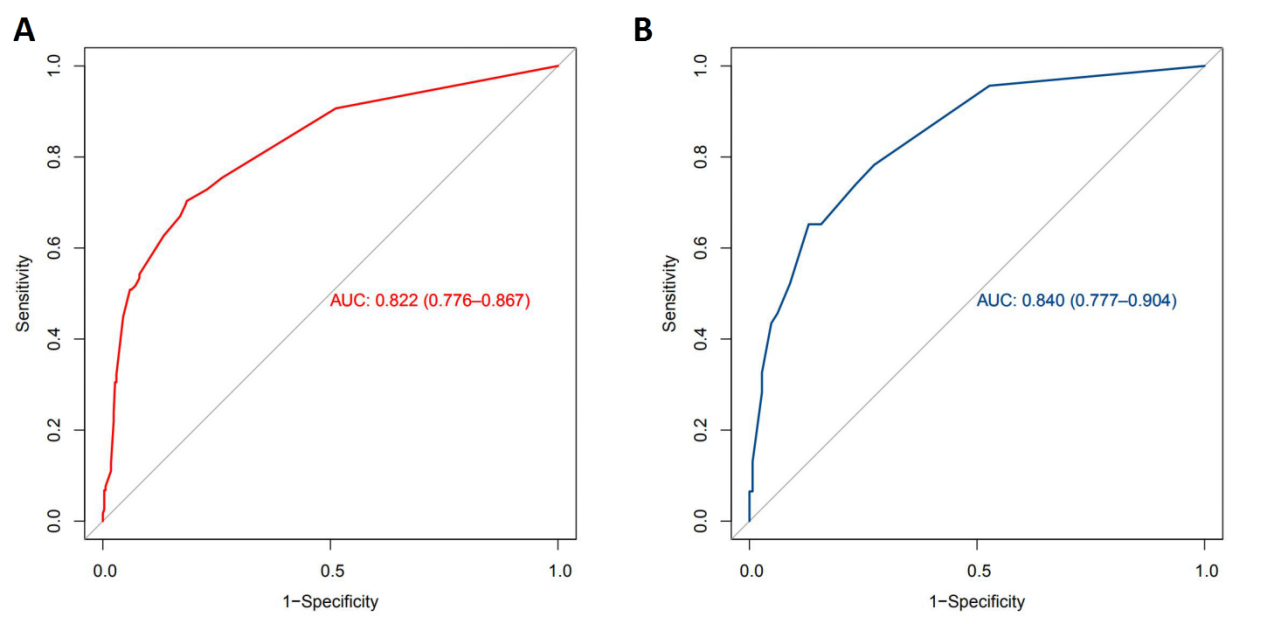  rm(list = ls(all = TRUE))  > ####Work Path Setting  > setwd("C:/Users/23983/Desktop/R results")  > ####Data import  > #It is recommended to use UTF-8 csv format to import data  > data <- read.csv("trainTotal data (+ disaggregated information) (10 risk factors).csv",header = TRUE)  > test <- read.csv("vadTotal data (+ disaggregated information) (10 risk factors).csv",header = TRUE)  > ###############Training set model training and probability derivation###############  > fit.full <- glm(END ~ V2+V3+V8+V17+V20+V21, data =test, family = 'binomial')  > fit.full <- glm(END ~ V2+V3+V8+V17+V20+V21, data =train, family = 'binomial')  > train$predict <- predict(object = fit.full, type = "response", newdata = train)  > test$predict <- predict(object = fit.full,type = "response", newdata = test)  > ###############Differentiation-ROC curve plotting###############  > library(pROC)  > roc.train <- roc(train$END,train$predict,ci=TRUE,polt=TRUE)  > plot(roc.train,  + col = 'red', #Setting the curve colour  + legacy.axes = TRUE, #So that the x-axis becomes1-Specificity  + xlab = '1-Specificity',  + print.auc = TRUE)  > roc.train  > roc.test <- roc(test$END,test$predict,ci=TRUE,polt=TRUE)  > plot(roc.test,  + col = '#00468BFF', #Setting the curve colour  + legacy.axes = TRUE, #So that the x-axis becomes1-Specificity  + xlab = '1-Specificity',  + print.auc = TRUE)  > roc.test |
| --- |
