# Supplementary material for: Easy-to-use nomogram to predict neonatal hyperbilirubinemia
Source: PeerJ. 2025 Sep 3;13:e20017. doi: 10.7717/peerj.20017 (PMC12422276; doi:10.7717/peerj.20017)
Supplement: Supplemental Information 4 [file peerj-13-20017-s004.docx]

FIGURE 4


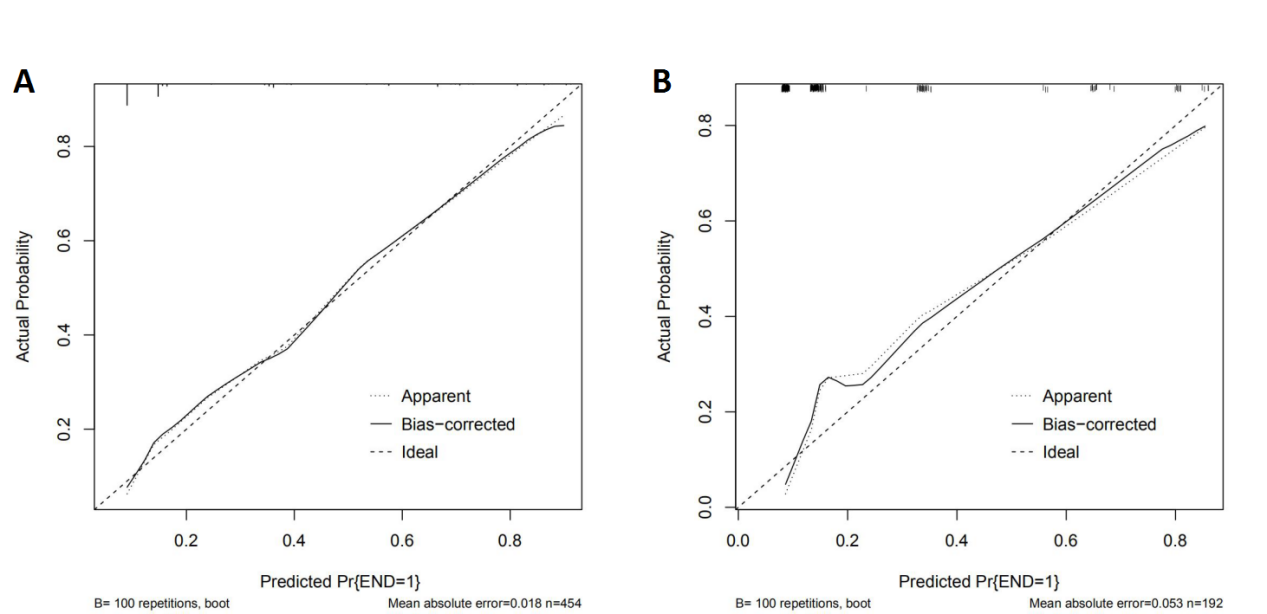


| > ###############Degree of calibration###############  > library(rms)  > set.seed(216)  > dist.train <- datadist(train)  > options(datadist="dist.train")  > predict.train <- lrm(END ~ predict, data = train,x = T,y = T)  > cal.train <- calibrate(predict.train, method="boot", B=100,add=FALSE)  > plot(cal.train)  > predict.test <- lrm(END ~ predict, data = test,x = T,y = T)  > cal.test <- calibrate(predict.test, method="boot", B=100,add=FALSE)  > plot(cal.test) |
| --- |
|  |
| \|  \| \| --- \| |
