# Supplementary material for: Easy-to-use nomogram to predict neonatal hyperbilirubinemia
Source: PeerJ. 2025 Sep 3;13:e20017. doi: 10.7717/peerj.20017 (PMC12422276; doi:10.7717/peerj.20017)
Supplement: Supplemental Information 5 [file peerj-13-20017-s005.docx]

FIGURE 5


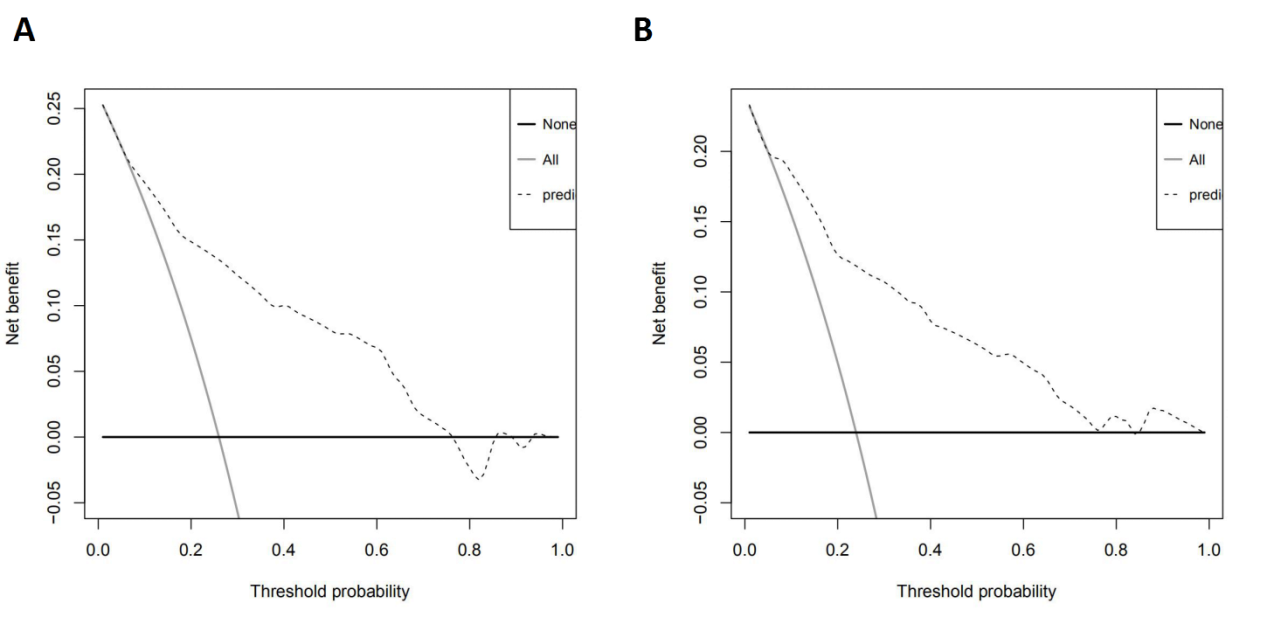


> ###############Decision curve###############

> source("dca.r")

> dca(data = train,outcome = "END",predictors = "predict",

+ smooth="TRUE",probability="TRUE")

> dca(data = test,outcome = "END",predictors = "predict",

+ smooth="TRUE",probability="TRUE")
