# Supplementary figures and images for: Easy-to-use nomogram to predict neonatal hyperbilirubinemia
Source: PeerJ. 2025 Sep 3;13:e20017. doi: 10.7717/peerj.20017 (PMC12422276; doi:10.7717/peerj.20017)

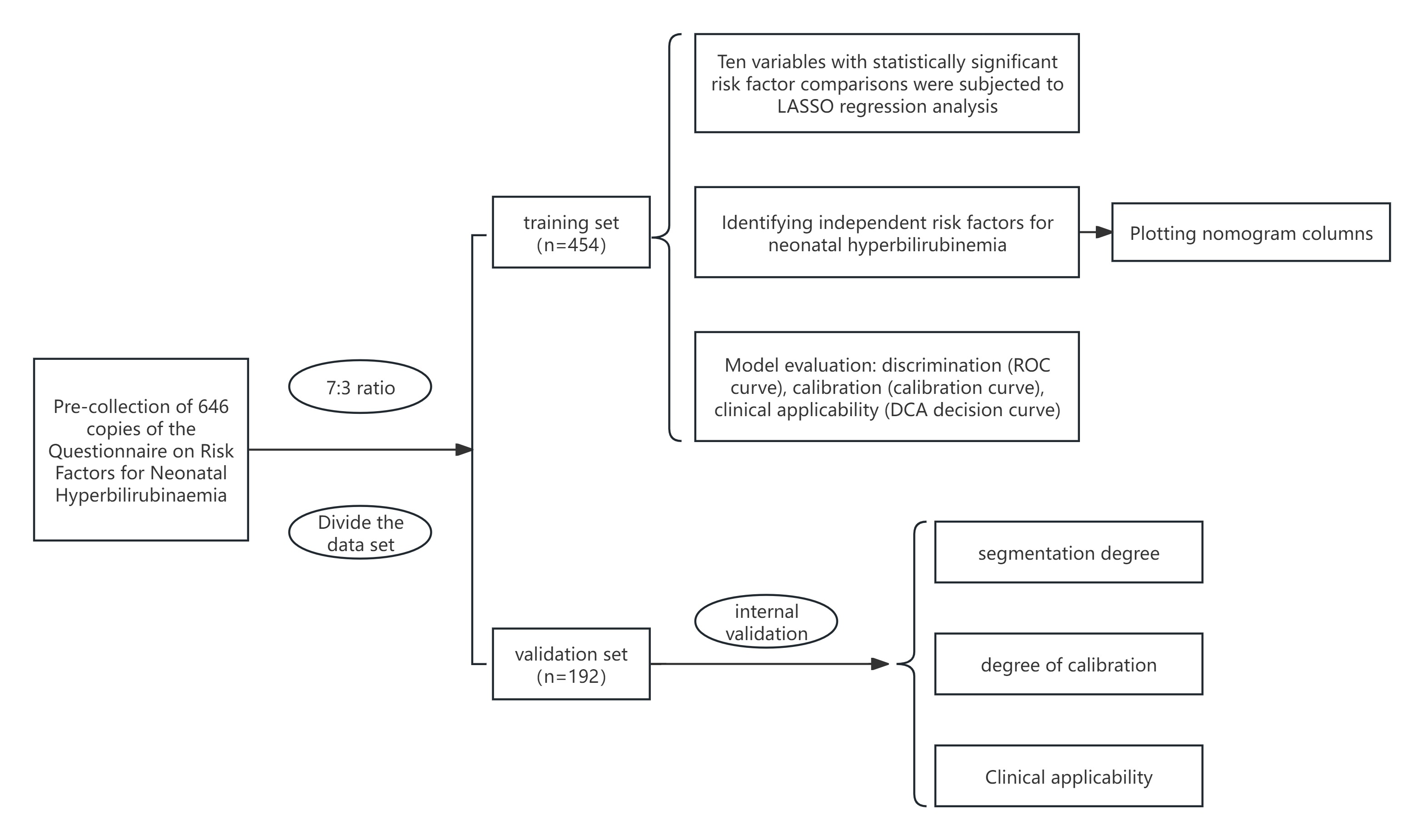

Supplement: Supplemental Information 6 [file peerj-13-20017-s006.jpg]
