# Supplementary material for: Easy-to-use nomogram to predict neonatal hyperbilirubinemia
Source: PeerJ. 2025 Sep 3;13:e20017. doi: 10.7717/peerj.20017 (PMC12422276; doi:10.7717/peerj.20017)
Supplement: Supplemental Information 7 [file peerj-13-20017-s007.docx]

Supplementary Materials


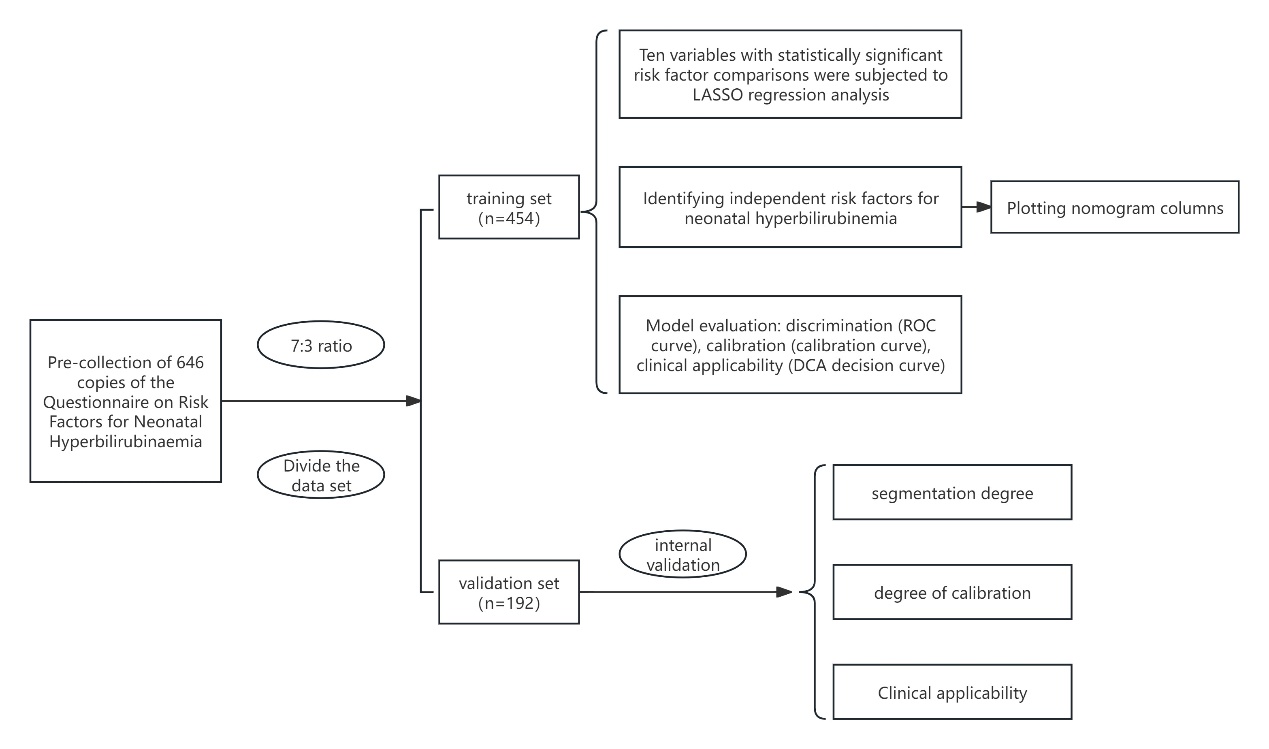
Figure S1 Flow chart of enrollment and follow-up evaluation of the development and validation datasets.

Table S1 Comparison of clinical characteristics between NNHB and NHB.

| Group | GA (w) | BW (g) | Gender | |
| --- | --- | --- | --- | --- |
|  |  |  | Male Female | |
| NNHB (n=482) | 38.72±1.12 | 3336.98±414.31 | 258 (53.53) | 224 (46.47) |
| NHB (n=164) | 37.54±1.66 | 3049.85±620.12 | 92 (56.10) | 72 (43.90) |
| t/χ^2^ | -10.230 | -6.690 | 0.230 | |
| *P* | < 0.001 | < 0.001 | 0.631 | |

Abbreviations: GA: gestational age; BW: birth weight; NHB: Neonatal hyperbilirubinemia; NNHB: Non-neonatal hyperbilirubinemia.

Table S2 Comparison of several factors between NNHB and NHB.

| Factor | NNHB(n=482) | NHB(n=164) | χ^2^ | *P* |
| --- | --- | --- | --- | --- |
| Umbilical blood PH ＜ 7.2,n (%) | 6 (1.24) | 3 (1.83) | 0.028 | 0.868 |
| Apgar scores ≤ 7, n (%) | 6 (1.24) | 5 (3.05) | 1.423 | 0.233 |
| Maternal-infant blood type, incompatibility and Coomb’ s test (+), n (%) | 21 (4.36) | 35 (21.34) | 42.466 | < 0.001 |
| Cephalohematoma, n (%) | 3 (0.62) | 2 (1.22) | 0.057 | 0.812 |
| Breastfeeding, n (%) | 473 (98.13) | 156 (95.12) | 3.233 | 0.072 |
| Probiotics, n (%) | 299 (62.03) | 48 (29.27) | 51.528 | < 0.001 |
| Weight loss ＞ 9%, n (%) | 2 (0.41) | 8 (4.88) | 13.199 | < 0.001 |

Abbreviations: NHB: Neonatal hyperbilirubinemia; NNHB: Non-neonatal hyperbilirubinemia.

Table S3 Maternal Clinical Characteristics Compared Between NNHB and NHB.

| Factor | NNHB (n=482) | NHB (n=164) | t/χ^2^ | *P* |
| --- | --- | --- | --- | --- |
| Maternal age (y) (Mean±SD) | 30.83 ± 3.56 | 31.39 ± 3.84 | 1.720 | 0.086 |
| Cesarean section, n (%) | 122 (25.31) | 63 (38.41) | 9.650 | 0.002 |
| Gestational hypertension, n (%) | 47 (9.75) | 31 (18.90) | 8.810 | 0.003 |
| Gestational diabetes, n (%) | 267 (55.39) | 74 (45.12) | 4.777 | 0.029 |
| Gestational hypothyroidism, n (%) | 21 (4.36) | 6 (3.66) | 0.026 | 0.873 |
| PROM ≥ 18h OR maternal fever, n (%) | 44 (9.13) | 44 (26.83) | 31.096 | < 0.001 |
| Placental abruption, n (%) | 8 (1.66) | 0 (0.00) | 1.566 | 0.211 |
| Placenta previa, n (%) | 9 (1.87) | 1 (0.61) | 0.579 | 0.447 |
| Prolonged labor, n (%) | 8 (1.66) | 4 (2.44) | 0.092 | 0.761 |
| meconium-stained amniotic fluid, n (%) | 62 (12.86) | 10 (6.10) | 4.993 | 0.025 |
| Abnormal umbilical cord, n (%) | 5 (1.04) | 4 (2.44) | 0.878 | 0.349 |

Abbreviations: NHB: Neonatal hyperbilirubinemia; NNHB: Non-neonatal hyperbilirubinemia.

Table S4 Binary logistic regression analysis of risk factors for neonatal hyperbilirubinaemia

| Factor | β | S.E. | Z | *P* | OR | 95%CI |
| --- | --- | --- | --- | --- | --- | --- |
| GA (w) | -0.498 | 0.105 | -4.710 | < 0.001 | 6.080 | 4.920~7.452 |
| BW (g) | -0.001 | 0.001 | -2.431 | 0.015 | 9.993 | 9.988~9.999 |
| PROM ≥ 18h OR maternal fever | 0.937 | 0.328 | 2.855 | 0.004 | 2.551 | 1.337~4.860 |
| Maternal-infant blood type, incompatibility and Coomb’ s test (+) | 2.077 | 0.395 | 5.257 | < 0.001 | 7.981 | 3.734~17.740 |
| Probiotics | -0.858 | 0.263 | -3.262 | 0.001 | 4.240 | 2.515~7.048 |
| Weight loss > 9% | 2.243 | 0.910 | 2.465 | 0.014 | 9.423 | 1.802~73.788 |

Abbreviations: GA: gestational age; BW: birth weight

Table S5 Assignment principle

| Factor | Group | Points |
| --- | --- | --- |
| GA (w) | 35 | 100 |
|  | 36 | 83 |
|  | 37 | 67 |
|  | 38 | 50 |
|  | 39 | 33 |
|  | 40 | 17 |
|  | 41 | 0 |
| BW (g) | 1000 | 91 |
|  | 1500 | 80 |
|  | 2000 | 68 |
|  | 2500 | 57 |
|  | 3000 | 46 |
|  | 3500 | 34 |
|  | 4000 | 23 |
|  | 4500 | 11 |
|  | 5000 | 0 |
| PROM ≥ 18h OR concurrent maternal fever | T | 31 |
|  | F | 0 |
| Maternal-infant blood type, incompatibility and Coomb’ s test | T | 70 |
|  | F | 0 |
| Probiotics | T | 29 |
|  | F | 0 |
| Weight loss > 9% | T | 75 |
|  | F | 0 |

Abbreviations: GA: gestational age; BW: birth weight; T: True; F: False
