# Supplementary material for: Easy-to-use nomogram to predict neonatal hyperbilirubinemia
Source: PeerJ. 2025 Sep 3;13:e20017. doi: 10.7717/peerj.20017 (PMC12422276; doi:10.7717/peerj.20017)
Supplement: Supplemental Information 8 [file peerj-13-20017-s008.docx]

Remarks:

END: 1 = hyperbilirubinaemia; 2 = non-hyperbilirubinaemia

GA (V2): 1=<38w; 2=≥38w

BW (V3): 1=<2500g; 2=≥2500g

Hypertension in pregnancy（V5）：1=Yes；0=No

Gestational diabetes (V6): 1=Yes; 0=No

Hypothyroidism in pregnancy（V7）：1=Yes；0=No

PROM≥18h OR maternal fever Maternal-infant blood type（V8）：1=Yes；0=No

Amniotic fluid situation (V12): 1 = clear; 2 = contaminated

Maternal-infant blood type Incompatibility AND Coomb’s test(+)（V17）：1=Yes；0=No

Probiotic supplementation（V20）：1=Yes；0=No

Weight loss >9% in 3 days（V21）：1=Yes；0=No

test: 1 = training set; 0 = validation set
